# Understanding the Impact of Ostomy Dejecta Constituents on Peristomal Skin Health and Models for Its Characterisation

**DOI:** 10.1111/iwj.70514

**Published:** 2025-05-21

**Authors:** Mirella A. Ejiugwo, Julie V. Gawenda, Abram D. Janis, Deborah A. McNamara, Sinéad T. O'Donnell, Shane Browne

**Affiliations:** ^1^ Tissue Engineering Research Group (TERG), Department of Anatomy and Regenerative Medicine Royal College of Surgeons in Ireland (RCSI) Dublin Ireland; ^2^ Hollister Incorporated Libertyville Illinois USA; ^3^ Department of Surgery Beaumont Hospital Dublin Ireland; ^4^ Department of Clinical Microbiology Royal College of Surgeons in Ireland (RCSI) Dublin Ireland; ^5^ Department of Clinical Microbiology Beaumont Hospital Dublin Ireland; ^6^ CÚRAM, Centre for Research in Medical Devices National University of Ireland Galway Ireland

**Keywords:** dejecta, ostomy, peristomal skin, peristomal skin complications, skin models

## Abstract

An ostomy, or stoma, is a surgically created percutaneous aperture from a hollow organ (e.g., small intestine) to the body's surface. Physicians may recommend an ostomy as a temporary or permanent solution to a range of disorders of the gastrointestinal tract, with up to 130 000 ostomies performed annually in the United States. An ostomy facilitates the expulsion of waste products, termed dejecta and circumvents the compromised organs. While an ostomy can be a lifesaving treatment, it is a disruption of regular digestive flow and has a number of associated complications including hernia, prolapse and necrosis. The most commonly observed complications are peristomal skin complications (PSCs), attributed to the leakage of dejecta onto the peristomal skin or the skin directly surrounding the stoma. Despite the prevalence of PSCs, little is known about the precise etiological factors that play a role in PSC formation. This review discusses the constituents of dejecta and their possible roles in PSC formation. Additionally, we identify a number of in vitro and in vivo skin models that could be used to study PSCs. Identification of the components of dejecta and understanding their interaction with skin models can facilitate the development of interventions to treat and prevent PSCs.

**Abbreviations**
2D: two‐dimensional
3D: three‐dimensional
ALI: air–liquid interface
BA: bile acid
BMI: body mass index
CFU: colony‐forming unit
DED: de‐epidermised dermis
ECM: extracellular matrix
GI: gastrointestinal
IAD: incontinence‐associated dermatitis
IBD: inflammatory bowel diseases
iPSC: induced pluripotent stem cell
LI: large intestine
PDMS: polydimethylsiloxane
PSC: peristomal skin complication
SI: small intestine
SOC: skin‐on‐a‐chip
TEWL: transepidermal water loss

## 1 Introduction

**Summary**

- An ostomy is a surgically created opening that allows waste to exit the body from a hollow organ, often used when parts of the gastrointestinal tract are compromised.
- While it can be life‐saving, it disrupts normal digestion and can lead to complications, particularly peristomal skin complications (PSCs) caused by waste (dejecta) leaking onto the surrounding skin.
- Despite their frequency, the exact causes of PSCs are not well understood.
- This review explores the constituents of dejecta and their potential role in PSC formation, highlighting skin models that can facilitate the development of interventions.

The digestive system plays a critical role in sustaining life, facilitating the breakdown of ingested macromolecules into simpler, absorbable compounds to support energy production, cellular maintenance and overall physiological function. However, several disease states disrupt the gastrointestinal (GI) system and compromise its function, including inflammatory bowel diseases (IBD), like Crohn's disease and ulcerative colitis, as well as diverticular disease and GI (colon and rectal) cancers. In these conditions, damage to the GI mucosa can cause pain, impair nutrient absorption and lead to serious complications such as malnutrition and anaemia. If left unmanaged, these diseases can result in abscesses, strictures, fistulas and even bowel perforation. Additionally, systemic effects may occur, including skin disorders, chronic liver disease, joint problems and vision impairment [1]. Taken together, these effects can have a profound impact on systemic health.

While some GI conditions can be managed with dietary adjustments, medications, or immunosuppressive therapy, others, particularly advanced GI cancers, may require surgical intervention [2, 3]. In cases where the inflammation cannot be adequately controlled or where the disease has caused irreparable damage to the GI tract, physicians may recommend surgery that sometimes includes an ostomy to restore function and improve quality of life. An ostomy, or stoma, denotes a surgically crafted opening from a hollow organ (e.g., the small intestine) to the body's surface, allowing for the elimination of waste products, which are collected in an ostomy bag. It is estimated that up to 1 million people live with an ostomy in the United States, with 100 000–130 000 ostomies performed annually [4, 5]. The European Union has approximately 700 000 ostomy patients, or ostomates, with these numbers expected to increase with the rising prevalence and incidence of IBD, among other GI disorders [6, 7].

**FIGURE 1:**
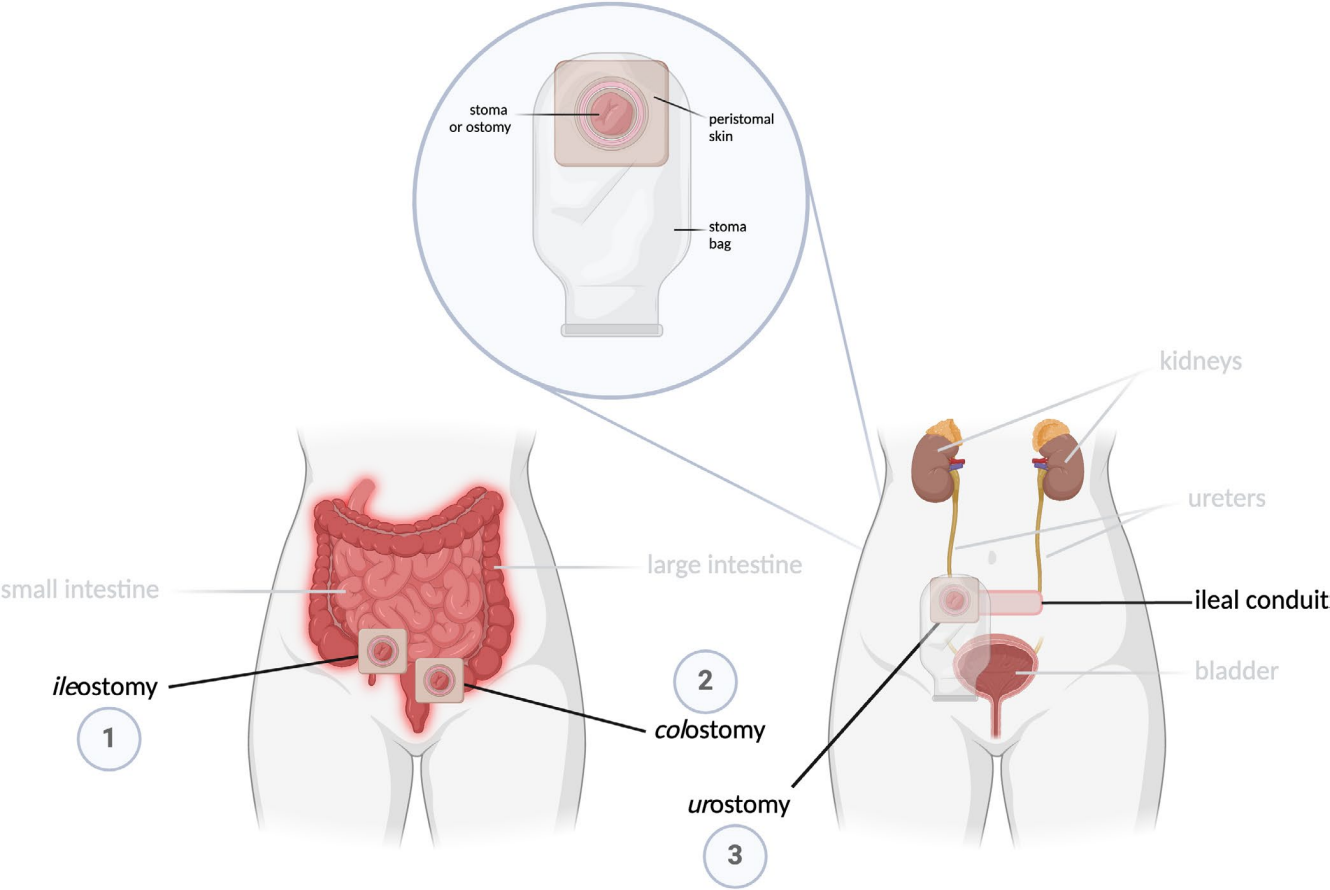
An overview of the various types of ostomies within the female anatomy. An ostomy bag is also shown, fitted over the stoma in the urostomy model. Created with BioRender.com.

An ostomy can be temporary or permanent, depending on the severity of the underlying condition. The suffix “‐ostomy” is derived from the Latin word *ostium*, meaning mouth or opening [8]. A prefix denotes the stoma's anatomical location, with three main types of ostomies depicted in Figure 1: (1) an *ile*ostomy, consisting of a diversion from the ileum of the small intestine; (2) a *col*ostomy, noted as a diversion of the large intestine or colon; and (3) a *ur*ostomy, involving surgically reattaching the ureters to a new reservoir, known as an ileal conduit, bypassing the bladder. The ostomy bag, attached to the abdominal surface, collects waste generically termed *dejecta*.

Ostomies represent a crucial treatment option for individuals suffering from diseases of the GI tract. However, a recent systematic review suggested an incidence rate of ostomates developing one or more complications up to 70% [3]. Ostomy complications are typically divided into early and late complications. Early complications, defined as complications within the first 30 days of surgery, include bleeding, hematoma, oedema, detachment, and necrosis. Late complications, usually classified as those occurring more than 30 days postoperatively, include high output, prolapse, stenosis and retraction [3, 9]. However, the most common complication, peristomal skin complications (PSCs), can occur at any time, with a reported prevalence ranging from 36.3% to 73.4% [5].

PSCs can manifest as irritation, inflammation, or infection around the stoma, representing a significant health and financial burden to ostomates. Ostomates with PSCs were significantly more prone to rehospitalization within 120 days post‐surgery (55.7% for ostomates with PSCs vs. 35.5% for ostomates without PSCs). Moreover, their subsequent hospital stays were lengthier; patients experiencing PSCs stayed an average of 11.0 vs. 6.8 days for those without PSCs [10]. Often, the PSC's physical appearance takes a mental toll, where ostomates with PSCs were shown to be 1.88 times more likely to have a negative body image than those without PSCs [11].

The spillage of dejecta onto the skin is a significant risk factor for PSCs [12, 13]; however, little is known about the interaction of dejecta with the skin. In an ileostomy and colostomy, the dejecta includes partially digested nutrients, bacteria, digestive enzymes and bile. These active constituents of GI dejecta, introduced to the skin through undermining the adhesive ostomy barrier or ostomy bag spillage, are suspected to be critical to PSC formation; however, there is a dearth of PSC‐specific models to evaluate specific causative factors. Therefore, this review will examine the active constituents of GI dejecta as potential contributors to PSCs and identify skin models that may be used to interrogate the constituents of dejecta and their role in PSCs. Urostomy dejecta is unlike other ostomy dejecta, being a diversion of the urinary tract rather than of the GI tract; hence, the focus of this review will be on PSC causation by ileostomy and colostomy dejecta. By elucidating the mechanisms underlying PSCs and exploring applicable features of diseased skin models, this review aims to highlight the path towards developing models of PSC. Such models can provide valuable insights into the pathogenesis of PSCs, leading to an increased mechanistic understanding of the causation of PSCs, ultimately facilitating the development of improved stoma care products, practices and interventions.

## 2 The Healthy Environment of the GI Tract

Knowledge of the small intestine (SI) and large intestine (LI) is essential to understand the role of dejecta as a function of the stoma site in the development of PSCs.

After ingestion of food, our digestive processes are activated, beginning with the mechanical grinding of food by the teeth and the release of salivary amylase and lingual lipase. The food is transformed into a bolus that is swallowed and pushed down to the stomach by the peristaltic motion of the oesophagus. In the stomach, gastric juice secretion, consisting of hydrochloric acid, gastric lipase and pepsin, degrades the bolus into chyme, a pulpy fluid, which enters the SI through the pyloric sphincter [14].

**FIGURE 2:**
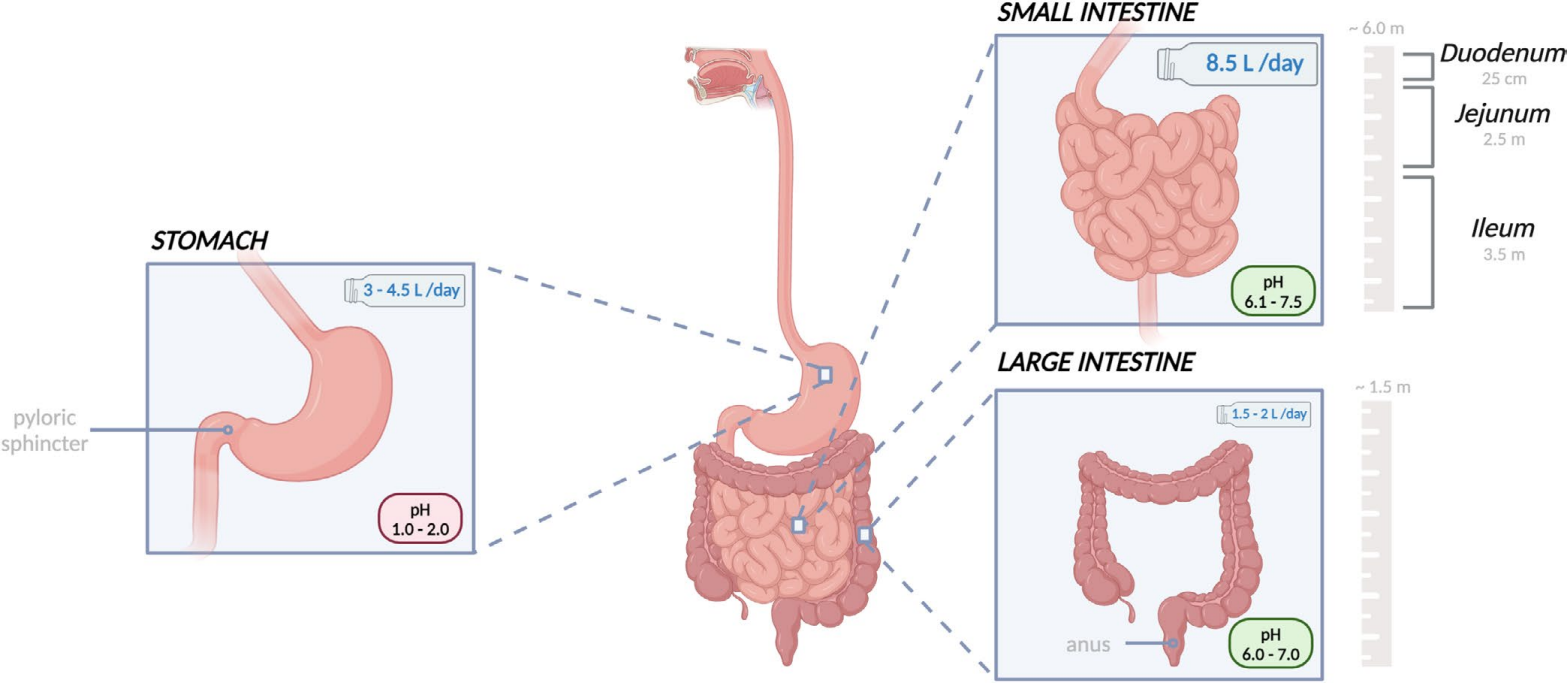
An overview of the GI tract, focusing on the stomach, small intestine and large intestine. The volume hosted daily by each organ is recorded in litres within the bottle symbol. Created with BioRender.com.

Contrary to its description as “small,” the SI is approximately 7 m long with a diameter of 3.5 cm, folded in the abdomen. The SI is subdivided into three sections, from proximal to distal: the duodenum, jejunum and ileum (Figure 2). The primary role of the SI is to absorb nutrients and water and transport chyme along the GI tract. In health, the SI is distensible and mobile within the abdominal cavity and its contents progress distally through movement known as peristalsis. The SI hosts a volume of 8.5 L of fluid daily, of which bile and pancreatic secretions (including active degrading enzymes) make up 0.5 L and 1.5 L, respectively. Only approximately 2 L are transported to the LI for further processing, and the remainder is reabsorbed by the SI [15, 16].

The duodenum (mean pH 6.1) is closest to the stomach and makes up approximately the first 25 cm of the SI [17, 18]. It receives chyme from the stomach, which is then neutralised by bicarbonate and mucus, and introduces hepatic bile and pancreatic secretions via the common bile duct. The secreted bile concentrations are toxic to microbes, while the pancreatic secretions are mixtures of digestive enzymes (proteases, glycosidases and lipases). Upon entry of chyme into the duodenum, hormonal glands initiate signals that release digestive juices (namely, pancreatic amylase, pancreatic lipase and pancreatic proteases). The duodenum is also the absorption site for iron and folate [18, 19].

The duodenum is followed by the jejunum (mean pH 7.1) that is roughly 2.5 m long. The physiology of the jejunum enables the absorption of 95% of carbohydrates, proteins, and 90% of water from the chyme [15, 19]. Following the jejunum is the ileum (pH 7.4–7.5), the most distal and extended section of the SI [17, 20] that ends in the right lower quadrant of the abdomen and is separated from the large intestine by the ileocecal valve. The transit time of chyme in the ileum is the longest of the SI. Vitamin B12, bile acids and nutrients not absorbed in earlier parts of the SI are absorbed in the ileum [15, 19, 21].

In contrast with the SI, the LI has lower enzymatic activity [22]. The LI begins at the ileocecal valve (pH 6.0–7.0) and ends at the anus, with a length of roughly 1.4 m in adults. The LI consists of the cecum, appendix, ascending colon, transverse colon, descending colon, sigmoid colon, rectum and anal canal. The key functions of the LI are water and electrolyte absorption, vitamin production and absorption, faecal formation and motility for excretion. Therefore, approximately 100–200 mL of fluid and 2–5 mEq of sodium are eliminated as faeces daily [17, 23].

## 3 The Burden of PSCs

Considering the anatomy, physiology and critical functions of the GI tract, one can understand the disruption caused by an ostomy. An ostomy precludes further digestion, preventing transformation of chyme to faeces, and instead excreting dejecta as the final waste product. Peristomal skin refers to the skin immediately surrounding the stoma, where the mucosa of the GI tract is surgically attached to the skin of the abdominal wall. This is the area where attachment of the adhesive component of the ostomy bag to the abdomen takes place, an area that is included in almost all PSCs. Such PSCs appear as visible irritation around the stoma with varying degrees of severity and encompass a range of specific conditions including allergic contact dermatitis, ulceration and fungal infection [24]. These complications can impact the epidermal and dermal layers of the skin. For example, peristomal pyoderma gangrenosum manifests as a reactive, non‐infectious inflammatory PSC characterised by a painful, red lesion that swiftly transforms into a blistered or necrotic ulcer in the peristomal skin area. These ulcers can penetrate through the epidermis and expand in size if not adequately treated [25].

Pre‐operative and post‐operative risk factors can increase an ostomate's risk for PSCs. Pre‐operative risk factors include correct selection of the stoma site, the nature of the surgery (emergency or elective), and the site of intestine exteriorised. In standard procedures, marking starts by locating the ‘ostomy triangle’, which is defined by the anterior superior iliac spine, the pubic tubercle and the umbilicus. The stoma, typically an ileostomy on the right and a colostomy on the left, is positioned in the centre of this triangle and should be at least 5 cm away from skin folds, previous scars, bony prominences and the patient's belt line. An elective operation, with carefully planned stoma placement and correct technical stoma formation, can reduce the risk of PSCs [26, 27]. Post‐operative risk factors include inexpert stoma care nursing and insufficient patient training in care of the skin surrounding the ostomy. Standardised ostomy education is essential: learning to empty the ostomy bag properly, disinfect the peristomal skin and avoid skin stripping can considerably decrease the risk of complications [28]. In addition to these risk factors, an individual's age, stoma type, body habitus, indications for surgery and adhesive skin barrier design can influence PSC formation [28, 29].

Yet, even with ideal stoma placement and ostomy aftercare, dejecta spillage may still occur. Persistent leakage complicates the secure attachment of the ostomy bag, which, in turn, leads to increased leakage. This creates a ‘vicious circle’ that propagates skin damage and PSCs, negatively impacting an ostomate's quality of life.

## 4 The Active Constituents of Dejecta Contributing to PSCs

Generally, the more distal the ostomy, the more well‐formed the excreted dejecta and the smaller the volume. The ileum absorbs bile salts and vitamin B12; thus, with an ileostomy, the recycling of bile salts decreases, correlating with a decrease in fat absorption in the jejunum. Additionally, the healthy ileum slows intestinal motility; therefore, SI transit time is significantly shortened by an ileostomy [30]. A typical ileostomy can produce 500–1500 mL of highly active, alkaline and semi‐fluid dejecta daily [3, 24, 28]. The composition of ileostomy dejecta is nearly 90% water, excreting elevated amounts of sodium (30–80 mEq) compared to 2–10 mEq of faecal sodium losses in a healthy individual [30].

The LI absorbs sodium and water; however, with intact ileum reabsorbing bile salts, absorption in the LI with a colostomy remains largely unaffected. Therefore, the consistency of colostomy dejecta is like normal defecation, with a daily production of 200–700 mL [24, 31, 32]. Typically, ileostomies and colostomies require a bag change at least twice a week with more frequent emptying or changes depending on output volumes and consistency.

**FIGURE 3:**
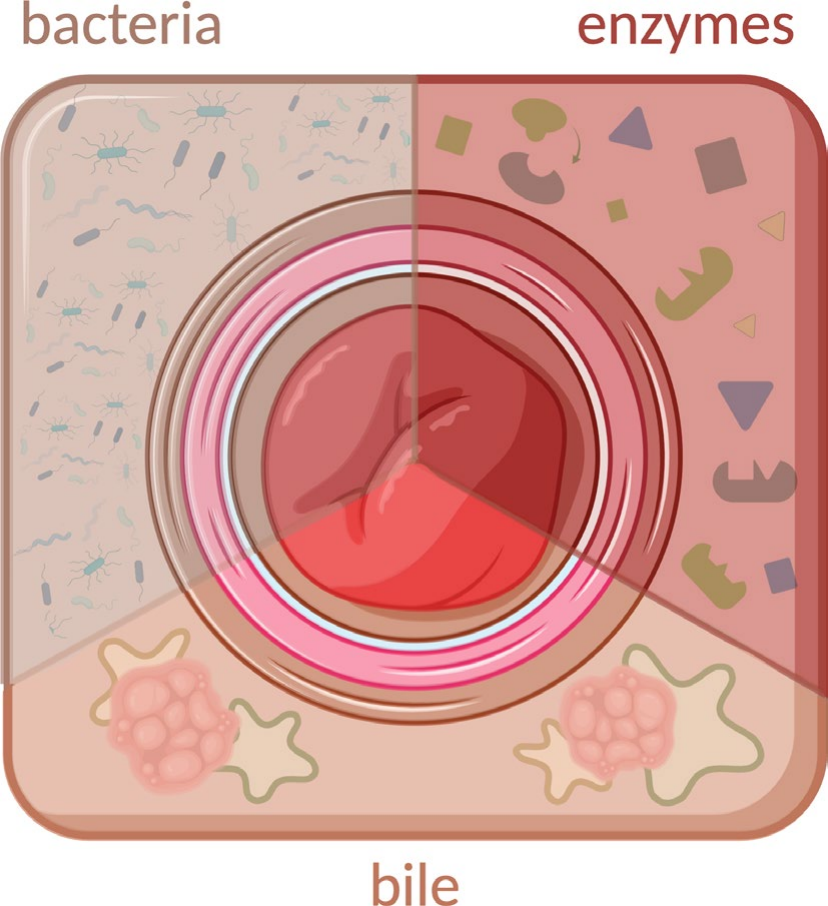
A stoma partitioned by the suspected PSC‐causing active constituents of dejecta (bacteria, enzymes and bile). Created with BioRender.com.

The interplay between the active constituents of dejecta, including bacteria, digestive enzymes (proteases, lipases, etc.) and bile, depicted in Figure 3, is suspected to be critical to PSCs. Healthy skin has an acidic mantle, ranging from pH 4.1 to 5.8, which is critical for maintaining its barrier function. Alterations to the skin's pH, such as dejecta leakage, could adversely affect lipid metabolism, which acts as a surrounding matrix for cornified cells [33]. The abdominal skinfold region, where an ostomy is created, constitutes a “physiological gap” and is characterised by an alkaline pH and is associated with an increased risk of skin barrier disruption, particularly for individuals with truncal obesity [34]. Additionally, multiple endogenous factors can alter the healthy pH of the skin, such as ageing, gender and underlying skin conditions that, acting synergistically with the active constituents of dejecta, may further erode peristomal skin [34, 35].

In an acute wound setting, a small volume of exudate aids tissue repair due to the presence of reparative growth factors. However, in the case of PSCs, there is a significantly higher exudate and dejecta volume, which can lead to skin maceration. Skin maceration refers to the softening and breakdown of skin due to prolonged exposure to moisture, leading to soft, pale and wrinkled skin [36]. The stratum corneum becomes moisture‐saturated and swollen, and the intercellular lipid lamellae in the corneal layer of the epidermis are disrupted. These events result in reduced epidermal barrier function, increased transepidermal water loss (TEWL) and transdermal penetration of solutes—further worsened by bacteria, enzymes, bile and their metabolites [37]—and forming PSCs. The following sections describe each constituent and its hypothetical contribution to PSC formation, summarised in Table 1.

**TABLE 1:** Summary of proposed effects of active dejecta constituents (bacteria, digestive enzymes and bile) on peristomal skin.

Dejecta constituents	Physiological function within the GI Tract	Potential effects on peristomal skin
Bacteria	Living microorganisms that contribute to carbohydrate and fat digestion, bile acid physiology and micronutrient synthesis and absorption.	**Dermis** Contribution to extracellular matrix (ECM) breakdown of the peristomal skin via collagenase synthesis and associated high activity by aerobes and facultative anaerobes. Additional breakdown via microbial‐induced matrix metalloproteinase (MMP) synthesis.
Digestive enzymes	Proteins responsible for the digestion of ingested food for nutrient absorption via circulation.	**Epidermis** Breakdown of the sebum and intercellular lipid lamellae within the stratum corneum by pancreatic lipase.
		**Dermis** Protease‐governed cleavage of ECM components within the skin. Cleavage of collagen, excessive desquamation and increased macromolecule transdermal penetration by chymotrypsin and trypsin. Elastase cleavage of elastin fibres.
Bile	Secreted by the liver. Involved in dietary fat emulsification and breakdown for improved digestion and absorption. Reabsorbed along the terminal ileum.	**Dermis** Bile acids (BAs) are used as transdermal‐enhancing excipients for drug formulation. BA exposure may enable increased entry of pancreatic enzymes and bacteria into the peristomal skin.

## 5 Bacteria

The gut microbiota consists of microorganisms (bacteria, fungi, viruses, etc.,) which are integral in regulating digestion, metabolism and immunity. Certain intestinal bacteria produce B vitamins, including thiamine (B1) and pantothenic acid (B5), which assist with food‐to‐energy conversion and neurotransmitter synthesis [38]. Other intestinal bacteria enable the biotransformation of bile, a crucial element in the enterohepatic circulation that assists with the absorption of dietary lipids and fat‐soluble vitamins [39]. Additionally, some of the microbiota population governs the development of the immune system, producing signals to control regulatory T cells and T helper 17 cells [40].

The gut microbiota is not homogenous along the GI tract, with the bacterial population varying from 10^1^ to 10^12^ colony‐forming units per millilitre (CFU/mL), shown in Table 2. The lack of homogeneity is compounded by significant interindividual diversity, with the Human Microbiome Project unveiling limited phylogenetic overlap among 242 healthy adult participants [47]. Several factors contribute to the uniqueness of gut microbiota composition, including age, diet, antibiotic usage and geographical location [48].

**TABLE 2:** A table summarizing the bacterial population, major genera, anaerobic versus aerobic bacterial dominance, and intraluminal pH, by GI segment (SI; small intestine, LI; large intestine).

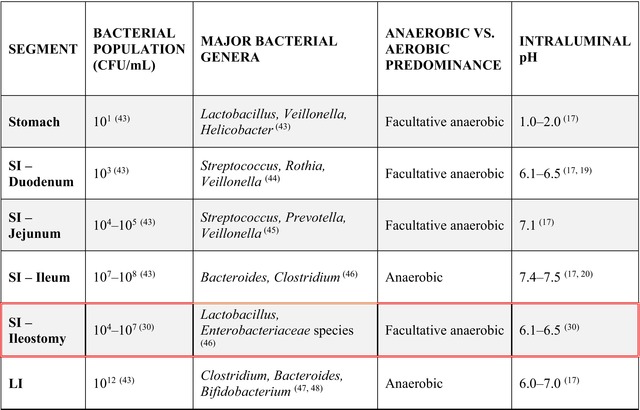

*Note:* The segments boxed in red signify the changes invoked in healthy segments due to an ostomy.

While each gut microbiome is unique, researchers have established connections between characteristics of gut microbial dysbiosis, imbalance, and/or reduced species diversity, and certain medical conditions, including interventions such as ostomies [44, 49, 50]. For instance, Crohn's disease is associated with an increase in specific bacteria, such as *Faecalibacterium prausnitzii* , alongside an overall reduction in bacterial diversity. Conversely, such a specific change in diversity is not typically observed in ulcerative colitis [51].

In the late 1960s, Finegold et al. described the ratio of anaerobes to aerobes within human ileostomy dejecta as 1:1000, while Ruseler‐van‐Embden et al. described the ratio of anaerobes to aerobes within experimentally ileostomised dog dejecta as 1:20 [49, 50]. Typically, the lower GI tract is dominated by anaerobic bacteria due to low oxygen levels caused by the rapid oxygen consumption by other microbes and host tissue. Yet, when the SI encounters oxygen through the stoma, it shifts the bacterial makeup, favouring facultative anaerobic bacteria over obligate anaerobes. Facultative anaerobes, which can thrive in both oxygen‐rich and oxygen‐deprived conditions, gain an advantage over obligate anaerobes. The presence of facultative anaerobes has been repeatedly confirmed, most notably by Hartman et al., where among 229 dejecta samples, facultatively anaerobic *Lactobacillus* species and members of the *Enterobacteriaceae* family dominated the bacterial population, an inversion of the typical anaerobic gut profile of *Bacteroides* and *Clostridia* species [44]. The major bacterial genera and their oxygen dependency are classified by GI segment in Table 2.

Additionally, an oxygen‐rich environment can stimulate the proliferation of specific microorganisms which may be implicated in PSCs. Aerobic bacteria such as *Bacillus subtilis* and facultative anaerobes such as *Enterococcus faecalis* produce collagenase enzymes that degrade intestinal tissues at a rate seven orders of magnitude higher than native intestinal collagenase. Moreover, these microbial‐derived collagenases stimulate the production of matrix metalloproteinase‐9 within intestinal tissues, a collagen‐degrading enzyme with intrinsic tissue‐destructive capabilities [52, 53]. These collagenases can be introduced to the skin through dejecta spillage, directly causing PSCs. Even relative proportions of short‐chain fatty acids, the end‐products of complex carbohydrates following bacterial metabolic activity, differ between “healthy” ileal effluent and ileostomy dejecta, respectively: acetate (60% vs. 78%), propionate (12% vs. 5%) and butyrate (18% vs. 17%) [54].

Notably, these gut microbial changes are not permanent. Studies have shown that upon reversal of an ostomy, the microbial community transitions back to a predominance of obligate anaerobes, highlighting the plasticity of the gut microbiome and its ability to adapt to changes in oxygen availability and other factors within the intestine [44].

## 6 Digestive Enzymes

The GI tract is functionally dependent on some key components necessary for the successful digestion and absorption of nutrients, while preventing and countering pathogen survival [16]. In the presence of slightly acidic chyme in the duodenum, pancreatic juices are released. Pancreatic juices are comprised of enzyme‐rich secretions (pancreatic proteases, pancreatic lipase and pancreatic amylase) and alkaline buffering bicarbonate, which provides an appropriate pH for activating pancreatic digestive enzymes, rendering it isotonic [55].

Pancreatic proteases are responsible for protein digestion and are secreted in their non‐active form (i.e., trypsinogen, chymotrypsinogen, procarboxypeptidase and proelastase). Enterokinase catalyses the conversion of trypsinogen into its active form trypsin. Trypsin, in turn, activates the other pancreatic proteases [56]. Among the pancreatic proteases, proelastase (“elastase” in its active form) accounts for about 6% of total pancreatic enzyme output. Interestingly, elastase is minimally degraded along the GI tract and remains chemically stable even in faeces for up to 7 days [57]. Hence, the presence of elastase in dejecta can contribute to PSCs by the hydrolysis of elastin fibres [58]. Additionally, trypsin‐ and chymotrypsin‐like proteases, involved in desquamation, are found in skin [59]. Resultant of their desquamatory abilities, the presence of these proteases on skin can also increase transdermal penetration of macromolecules [60]. Therefore, the increased presence of these proteases due to dejecta spillage could lead to skin damage and the formation of PSCs.

On the other hand, pancreatic lipase emulsifies fats for SI absorption via the cleavage of fatty acid ester bonds in triglycerides and is stabilised by colipase and bile acids [55]. The sebum, or the most exterior layer of the skin, is composed mainly of triglycerides (lipids) to avoid water loss. Within the stratum corneum, the layer of skin below the sebum, intercellular lipid lamellae are likewise digestible by lipases. Hence, spillage of pancreatic lipase‐containing dejecta could lead to the breakdown of the sebum and intercellular lipid lamellae of the peristomal skin, progressively leading to the formation of PSCs [61]. Lastly, pancreatic amylase acts by cleaving glycosidic bonds to break down starch into smaller carbohydrate molecules that undergo hydrolysis in the duodenum and the jejunum, resulting in absorbable glucose, galactose and fructose [56]. While pancreatic amylase will not directly contribute to skin degradation, it can contribute to the alkalinity of the pancreatic secretions contained in the dejecta. Healthy skin ranges in pH from 4.1 to 5.8 [33]; thus, introducing a mixture of alkaline digestive enzymes through dejecta spillage to the acidic surface of the skin can weaken the skin barrier, making it more prone to enzymatic damage and PSCs.

## 7 Bile

Bile is a complex secretion of bile salts, bilirubin, phospholipids, fatty acids, cholesterol, electrolytes and water [62]. A daily volume of 600–750 mL of bile is secreted by the liver and, when not needed, is stored in the gallbladder [63]. Prior to excretion from the liver, the primary bile acids (BAs) cholic acid and chenodeoxycholic acid are conjugated to either glycine or taurine for improved solubility, reduced BA toxicity and better fat emulsification. Bile enters the duodenum via a common bile duct with pancreatic digestive juices [62].

The primary GI‐related functions of bile are: (1) to emulsify dietary lipids and fat‐soluble vitamins in the SI before the activity of the hydrophilic pancreatic lipase for better digestion and absorption in the SI; (2) to eliminate waste products from the body, such as cholesterol and bilirubin [62]. The terminal ileum is the reabsorption site for 90%–95% of BAs [63]. Primary BAs that are not absorbed in the ileum are transformed by bacteria in the colon, hence forming the secondary BAs: deoxycholic acid, lithocholic acid and ursodeoxycholic acid. These latter substances are partly absorbed in the colon or excreted in the faeces [64].

Due to local inflammatory changes in the gut, individuals with IBD tend to have dysbiotic gut microbiotas compared to healthy individuals. In fact, isolates of *Fusobacterium varium* and *Escherichia coli* from IBD patients have successfully induced experimental colitis in mice [65, 66]. Coupled with dysregulated production of gut‐protective metabolites such as BAs and short‐chain fatty acids, dysbiosis in IBD contributes to altered gut barrier integrity [67, 68]. Furthermore, BA malabsorption is common in IBD patients, especially those with ileocolic disease, coupled with reduced colon activity, resulting in decreased secondary BA production. Hence, the altered gut microbiota in IBD and the associated reduced gut barrier integrity can impair bile acid metabolism and prove to be damaging to peristomal skin in the event of dejecta spillage [67].

Ileostomy formation can cause BA diarrhoea, leading to decreased reabsorption of conjugated BAs and fat malabsorption. Conjugated BAs are primary BAs that have been modified by the liver through attachment of glycine or taurine. As a result of decreased reabsorption, conjugated BAs are released as part of the dejecta, with loss of BAs in ileostomy dejecta increased up to 30% when compared to faecal BAs, especially when dietary protein and fat intake is high [69, 70]. Though alkaline and antimicrobial, primary BAs have been recently repurposed as transdermal‐enhancing excipients in formulating hydrophilic and hydrophobic drugs, especially cholic acid [71]. This characteristic confirms its ability to breach peristomal skin barrier integrity, especially through synergy with the other constituents in the dejecta. On the other hand, dejecta arising from a colostomy are more solid and contain less BAs. Therefore, direct contact of peristomal skin with colostomy dejecta may be less irritating than ileostomy dejecta [9].

## 8 Existing Models for Studying PSCs

**FIGURE 4:**
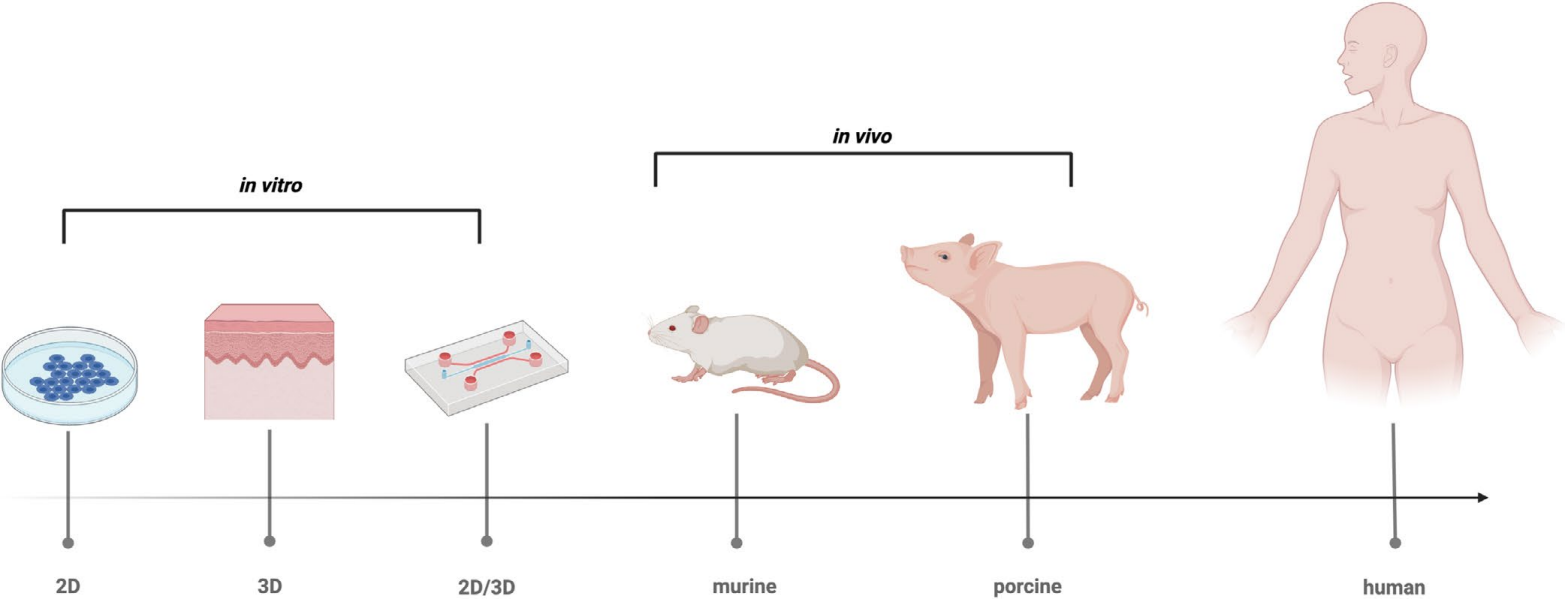
A graphical summary depicting in vitro and in vivo models that could be used to study PSCs and PSC‐like skin disorders, ranging from 2D in vitro systems to human volunteers. Created with BioRender.com.

Given the wide prevalence of PSCs, models are required to study PSCs and elucidate the mechanisms involved. A wide range of approaches exists that have been used to study various skin conditions, from simple 2D in vitro systems and 3D in vitro skin models, including skin‐on‐a‐chip, to in vivo models, shown in Figure 4. This section will review the available models and discuss their applicability and potential relevance to PSCs.

## 9 In Vitro

Early in vitro human skin models usually comprised a single cell layer, often keratinocytes, in a monolayer [72]. Co‐culture models consisting of multiple cell types (keratinocytes, fibroblasts, etc.) increased the complexity and relevance. These two‐dimensional (2D) models are still used to model various skin diseases and injuries, like psoriasiform cutaneous inflammation, which was induced in a 2D monolayer of keratinocytes via a cytokine cocktail of IL‐17A, IL‐22, IL‐1α, TNF‐α and oncostatin M [73]. Similarly, Jiao et al. co‐cultured human dermal fibroblasts with basophils and eosinophils and exposed them to NOD2/TLR2 ligands to investigate atopic‐dermatitis‐related pro‐inflammatory cytokine/chemokine expression [74]. Assembling 2D models is simple and cost‐effective, but unrepresentative of real skin structure. Additionally, the systems with only one cell type are overly simplistic, mimicking either the dermis or the epidermis, but not the interaction between the cell types involved. While co‐culture models increase complexity, they fail to recapitulate the spatiotemporal dynamics of cell‐to‐cell communication due to a lack of dimensionality.

**FIGURE 5:**
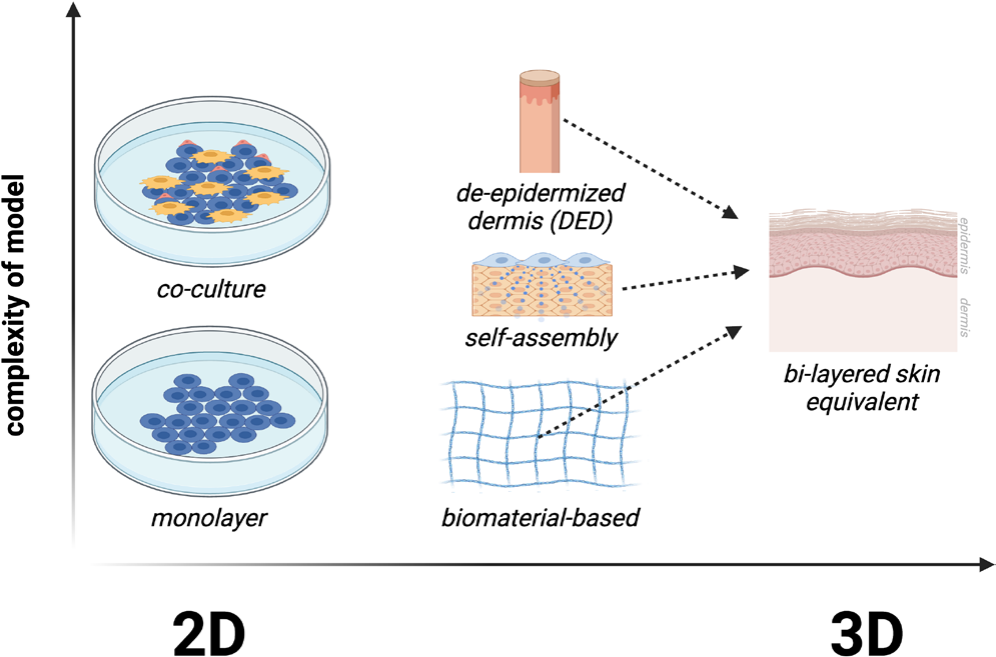
A graphic of the evolution of in vitro diseased skin models from 2D to 3D. Created with BioRender.com.

Three‐dimensional (3D) models replicate the skin structure in a more relevant manner. Typically, in 3D models, fibroblasts are first cultivated, followed by keratinocytes, to create the dermis and epidermis, respectively. The evolution of in vitro models, from 2D to 3D, is illustrated in Figure 5. 3D skin models can be categorised as de‐epidermised dermis (DED), biomaterial‐based, or self‐assembly. Additionally, in 3D models, an air–liquid interface (ALI) is employed during the cultivation of the epidermis, where the top layer is exposed to air to induce stratification of epidermal keratinocytes, forming multiple cell layers including basal, spinous, granular and cornified [75].

The DED model utilises dermis biopsies from human donors. After a biopsy, the dermis is sterilised via gamma irradiation, glycerol, or ethylene oxide and de‐epidermised. Then, the DED serves as a scaffold upon which cells are seeded. Fibroblasts are seeded to form the dermis on the subcutaneous side of the DED, and on the opposite side, keratinocytes are seeded to form the epidermis [76]. This approach has been used to study several chronic inflammatory skin disorders, including parakeratosis and psoriasis. Parakeratosis, the abnormal retention of nuclei within the stratum corneum, was induced within a DED skin model by saturating the media with pan‐transglutaminase inhibitors (putrescine, NTU283 and NTU285) [77]. Similarly, psoriasis‐like phenotypes were induced by stimulating DED skin models with cytokines including IL‐1α, TNF‐α and IL‐6 during the last 4 days of ALI culture [78]. While similar disease phenotypes were induced, the de‐epidermisation process between models differed, as there is no standardised approach. Additionally, the utilisation of biopsies from live donors can involve unnecessary surgical risks, while the quality and viability of dermal tissue obtained from deceased donors may be dependent on factors such as post‐mortem interval and preservation methods. Therefore, data may not be comparable across DED models due to variability of preparation techniques and biopsy sources.

Another approach involves encapsulation of fibroblasts within an extracellular matrix (ECM)‐mimetic biomaterial to form the dermal layer. Typically, collagen is used as the encapsulating biomaterial since it is the most abundant protein in the native ECM, providing structural and biological support to cells. Then, keratinocytes are seeded atop this dermal layer and cultured via ALI for epithelial stratification. A collagen‐based model of psoriasis was created by harvesting fibroblasts and keratinocytes from skin biopsies of patients with active chronic plaque psoriasis. This model reproduced key aspects of the psoriatic phenotype, such as the expression of pro‐inflammatory cytokines (TNF‐α, IFN‐γ and IL‐6) and the presence of the chemokine receptor CXCR2. However, inter‐donor variation was observed among the psoriatic models, typical of techniques requiring donor tissue [79].

A subsequent collagen‐based model reproduced the clinical hallmarks of eczematous dermatitis (keratinocyte apoptosis, spongiosis and chronic pruritus) by topical application of activated memory/effector (CD45RO+) T cells through the ALI media, in the last 12 days of cultivation [80]. However, the model utilised HaCaTs, an immortalised keratinocyte cell line, which is not entirely representative of human keratinocytes, having a different gene transcriptional profile of cornified envelope‐associated proteins (e.g., filaggrin and loricrin). This difference influences barrier development in chronic skin inflammatory disorders like dermatitis [81]. On the other hand, researchers have reported that patient‐derived cells have a limited capacity for subculturing, as from passage 3, keratinocytes derived from diabetic patients started showing differentiating phenotypes. This included becoming large‐sized, reduced proliferation and unexpectedly detaching from culture plates, with similar observations of diabetic fibroblasts from passage 6 onwards [82]. Additionally, collagen‐based models can vary depending on the source of collagen, which can impart variability [83]. For example, the properties of animal collagen, typically sourced from rat tail [79, 80] or bovine skin [82], can vary significantly in yield, denaturation temperature, and resistance to enzymatic degradation [84].

Self‐assembly approaches remove the need for an exogenous ECM or skin biopsies by relying on the ability of fibroblasts to deposit their own ECM. Fibroblasts cultured for 1–3 weeks aggregate and form dermal sheets which can be manually superimposed (e.g., in multiple layers). Alternatively, fibroblasts may be cultured in media supplemented with ascorbic acid to enhance ECM deposition. Then, keratinocytes are seeded across this self‐assembled dermis to form the epidermis, followed by ALI culture.

Psoriasis‐ and atopic dermatitis‐like phenotypes were induced in a self‐assembled skin model using dermal fibroblasts and primary keratinocytes from juvenile foreskin. The topical application of media infused with Th1 and Th17 cytokines induced psoriasis, while atopic dermatitis was induced by the further addition of Th2 cytokines. The resulting diseased skin models displayed impaired barrier function, increased inflammation, parakeratosis, hyperproliferation of keratinocytes (psoriasis‐specific trait), and apoptosis [85]. Another self‐assembled psoriatic skin model was developed using isolated psoriatic T‐cells activated by a mixture of phorbol 12‐myristate 13‐acetate and ionomycin. In this model, primary fibroblasts supplemented with ascorbic acid formed dermal sheets. Then, T‐cells were seeded across the surface of one sheet, while primary keratinocytes were seeded across the surface of another. After another week of culture, the sheets were stacked together, with the keratinocytes at the topmost layer, and cultured via ALI for another 3 weeks. This model, integrating the diseased aspect rather than inducing it through a topical application, successfully stimulated the production of IL‐17A, hyperproliferating keratinocytes, and expressing known psoriatic differentiation markers [86]. Despite these promising results, the self‐assembly approach is difficult to control, as the lack of a structure leads to the formation of irregular dermal sheets.

More recently, skin‐on‐a‐chip (SOC) models have been developed, which amalgamate the fundamental aspects of 2D and 3D skin models. The 2D element, akin to traditional cell cultures, involves a flat surface with lateral fluidic movement, while the 3D facet introduces depth by incorporating microfluidic channels that supply nutrients [87]. Typically, a SOC is fabricated by layering polydimethylsiloxane (PDMS) between a glass slide and a porous membrane, creating upper and lower chambers. The microfluidic channels are designed in the lower PDMS layer to facilitate the flow of a culture medium to the 3D skin equivalent placed in the culture chamber above the porous membrane [88, 89, 90].

One SOC modelled atopic dermatitis, where the upper PDMS chip layer contained a cylindrical culture chamber with a collagen‐based skin equivalent constructed of human dermal fibroblasts and primary keratinocytes. During the ALI culture of the collagen‐based model within the SOC, recombinant human IL‐4 and IL‐13 protein treatment induced atopic dermatitis [89]. Another SOC simulated inflammation by separating the cell culture chamber using porous membranes, allowing the co‐culture of different cell types (keratinocytes, fibroblasts and endothelial cells) in three separate PDMS chip layers. Oedema was induced by perfusion of TNF‐α through the middle fibroblast layer [90]. Thus, SOCs allow researchers to induce and study skin disorders within a controlled microenvironment; however, the initial generation of these models is time‐consuming and requires an in‐depth knowledge of microfabrication.

Although there is a deficiency of in vitro models exploring the pathophysiology of PSCs, the diseased skin models described can establish the groundwork for a skin model that recapitulates key aspects of PSCs. After all, the pathophysiology of PSCs is akin to phenotypes seen in diseases like dermatitis (atopic and eczematous), psoriasis, parakeratosis and diabetes. DED models, composed of decellularized dermal matrices, maintain native ECM composition and structure, but rely on the availability of suitable donor tissue (e.g., biopsy from an ostomate with active PSCs). Biomaterial‐based models offer tuneable properties, allowing for customisation and controlled manipulation of the diseased microenvironment, but typically lack complexity (i.e., typically composed of only 1–2 ECM components). Self‐assembly models enable cells to organise and interact for native‐like tissue organisation and cellular crosstalk but require precise control over cellular interactions and environmental conditions. SOC models integrate microfluidic technologies, offering controllable platforms for studying disease mechanisms despite their time‐consuming and complex generation. Overall, the choice of in vitro model depends on the specific research objectives, the complexity of the disease(s) being studied, and the desired applications.

## 10 In Vivo

Although there is no established in vivo model for PSCs, murine (small) and porcine (large) animals are commonly utilised for modelling skin‐related pathologies. Pigs are widely accepted as the optimal model for wounds and skin conditions due to the similarity in skin anatomy and physiology with humans, including wound healing mechanisms [91].

However, despite the significant difference in physiology, murine models are often used owing to cost and practicality. Therefore, in the context of PSCs, mice can model peristomal dermatitis, initially characterised by pruritus and erythema and in severe cases, progressing to lesions and ulceration of the peristomal skin. Peristomal dermatitis most likely occurs due to direct contact with dejecta, likened to the direct contact of urine, faeces and bacteria with surrounding skin in incontinence‐associated dermatitis (IAD) but is sometimes indistinguishable from allergic reactions to appliances and adhesives [92, 93]. IAD development arises from skin maceration and is further accelerated by exposure to urine and/or faeces in occluded skin. IAD is characterised by skin redness and can be accompanied by skin damage or secondary infection like PSCs. Hence, synthetic urine and protease‐containing agarose gels have been successfully used to mimic IAD in mice [93, 94, 95]. Proteolytically treated murine skin (using agarose gels containing proteases) is characterised by skin flare‐up, redness, inner tissue damage, increased skin hydration and TEWL compared to non‐treated counterparts. These traits are indicative of skin maceration and impaired skin integrity. However, in the absence of skin maceration, tissue damage occurred from the surface of the proteolytically treated skin [95].

*Pseudomonas aeruginosa* is present in the intestinal tract, the skin and the perineal area of IAD‐affected individuals. It is commonly used as a bacterial representative in in vivo models of IAD, given its varied motility patterns (swimming, swarming and twitching) alike to those observed in intestinal bacteria [95]. When proteolytically treated and macerated skin is coupled with *P. aeruginosa* inoculation, the epidermis undergoes digestion in the stratum spinosum and immune cell infiltration across the dermis. These characteristics make it different from irritant contact dermatitis (typically limited to the epidermis and the dermo‐epidermal junction). When coupled with bacterial inoculation, skin flare‐up is observable macroscopically, while bacterial clusters and inflammatory cell infiltration are identified within the epidermis and the papillary dermis histologically. In the absence of bacteria, the presence of proteolytic enzymes did not affect histological alteration to the murine skin; however, antigen‐presenting cell infiltration deep into the dermis occurred. It could be suggested that severe skin damage observed in IAD is the synergy of increased permeability due to the proteolytically macerated skin state and bacterial expression of virulence factors (e.g., proteases and lipases), resulting in significant skin tissue degradation from inside the skin.

Another study examined the contribution of lipidolytic enzymes to IAD development using ex vivo skin tissues from 6‐month‐old Sprague–Dawley rats. Following skin maceration treatment, in the presence of lipases and proteases, erythrocyte leakage (speculative of blood vessel damage and subsequent bleeding) was observed significantly deeper in the skin surface compared to protease‐only treated ex vivo rodent skin tissue counterparts. Blood vessel damage was hypothetically attributed to phospholipases acting on the phospholipid bilayer found on the erythrocyte cell membranes. When the same treatment plan was repeated in vivo, erythrocyte leakage in the papillary dermis and around the hair follicles was observed. Both experiments could suggest the synergetic action of lipases and proteases in disrupting skin structure. In fact, bleeding was not observed when only lipases were applied to macerated skin. This study concluded that, though lipases may not cause histological changes in the skin, they could quicken the entry of proteases across the macerated skin by disrupting intercellular lipid lamellae, resulting in skin damage [61].

Interestingly, the most relevant murine study to PSCs observed the formation of peristomal dermatitis, associated with the leakage of dejecta, on the peristomal skin of experimentally ileostomised rats. The dermatitis on rat skin was characterised by oxidative stress, significant epidermal and hair‐follicle atrophy, collagen loss and reduced angiogenesis and cell proliferation [96]. However, besides this example, there is a void of dejecta‐induced PSC modelling in mice and rats.

Dermatitis has also been successfully modelled in pigs using different biochemical or chemical solutions [97, 98, 99]. The most common chemical irritant, 2, 4‐dinitrochlorobenzene, is used to induce dermatitis by topical application to dorsal porcine skin during both the sensitization and challenge phases [98, 100, 101]. Indicators of IAD‐derived inflammation can be verified ranging from qualitative methods (e.g., fluorescent dye transdermal penetration, histological analysis and a visual scoring system whereby 0 indicates “absence of erythema” and 4 indicates “severe erythema”) to quantitative ones (e.g., TEWL and proinflammatory cytokine quantification) [102].

Unlike 2,4‐dinitrofluorobenzene, ovalbumin can induce atopic dermatitis in pigs without physically disrupting the skin barrier. Ovalbumin tends to trigger a dominant Th2 response like human atopic dermatitis. Following ovalbumin exposure, epidermal hyperplasia, oedema formation and infiltration of eosinophils and CD4+ lymphocytes are observable. Correlation is further detectable between local inflammation and systemic inflammation, marked by increased CD4+ lymphocyte count and increased gene expression levels of IL‐4, IL‐13 and IFN‐γ [103].

Dermatitis has also been successfully induced by vaporised sulphur mustard, a chemical warfare agent, in pigs. With this induction method, typical traits of dermatitis were observed, such as severe erythema and oedema [104]. Superantigen‐induced dermatitis involves using superantigens for inducing dermatitis such as staphylococcal enterotoxin B, since more than 90% of atopic dermatitis‐affected regions in patients are colonised by the superantigen‐producing *Staphylococcus aureus* . Superantigens stimulate immune cells to produce immunoglobulin‐E antibodies against the invading bacteria in individuals with dermatitis and can be administered either topically or intradermally. Staphylococcal enterotoxin B‐induced skin inflammation is characterised by localised erythema, increased blood flow in the skin, and oedema. Outstanding advantages of the superantigen‐induced dermatitis porcine model are high reproducibility and similar drug efficacy in individuals with atopic dermatitis, and its usefulness for anti‐inflammatory drug testing [105]. A handful of biochemical and chemical irritants used for inducing dermatitis in murine and porcine models are presented in Table 3.

**TABLE 3:** Common irritants used to induce dermatitis in murine and porcine models.

Biochemical irritants	Chemical irritants
Pancreatin (pH 9) [97, 102]	2, 4‐Dinitrochlorobenzene [98, 100, 101]
Proteolytic solution (trypsin and chymotrypsin mixture) [93, 95]	2,4‐Dinitrofluorobenzene [99, 103]
	Synthetic human urine (distilled water, urea, sodium chloride, magnesium sulphate and calcium chloride) [94]
Lipidolytic solution (lipases and phospholipase A2 mixture) [61]	Sulphur mustard [104]
	Ovalbumin [103]

Additionally, psoriasis has been successfully induced in mice and pigs. Topical delivery of imiquimod to the dorsum or/and ears of mice for up to 25 days has been used to mimic human psoriasis [106, 107, 108]. Likewise, psoriasis‐like skin has been successfully induced in pigs using propranolol topically. The most common site of irritant application is the porcine skin of the back ear, and between 8 and 14 days are required to establish the psoriatic model. Porcine models of psoriasis are characterised by psoriasis‐related manifestations: erythema, swelling, the presence of silvery lesions, increased expression levels of STAT3, VEGF and T helper 17 cells; parakeratosis and hyperkeratinisation within the epidermis [109, 110]. Existing pig models of dermatitis can serve as a benchmark for inflammatory‐related analyses in evaluating peristomal inflammation‐induced changes of the skin following an ostomy.

Given the predominance of diabetes, obesity and high body mass index (BMI) in ostomates that develop PSCs, inducing these conditions in murine or porcine models could enhance the relevance of any PSC model [111, 112, 113]. Furthermore, pre‐existing skin conditions like atopic dermatitis and psoriasis increase one's chance of developing a PSC following an ostomy [114]. For example, filaggrin mutations in atopic dermatitis‐affected skin result in increased pH and activation of skin proteases, which can impair skin barrier integrity even prior to dejecta exposure [115]. In terms of the elderly‐afflicted, the decreased filaggrin content and increased pH of aged skin can render it prone to damage following exposure to ileostomy dejecta [116]. Bacterial and fungal infections can likewise contribute to PSC formation, which has been modelled in vivo. For example, a study reported increased penetration of hydrocortisone into porcine skin in the presence of *Lactobacillus* species‐derived biosurfactants. These latter tend to remain stable and effective, even under extreme pH, temperature and salinity conditions and, in fact, are proposed as potential green permeation enhancers for drug formulation [117]. Therefore, gastric bacteria‐produced factors present within the dejecta could enhance transdermal penetration, accelerating the onset of PSCs. Collectively, these insights alongside knowledge from previous in vivo models of IAD and psoriasis can prove helpful in studying PSC pathogenesis using animal models.

## 11 Conclusion

PSCs are the most common ostomy‐related complication, yet the precise aetiology is not yet understood and has not been fully explored. Instead, stoma care research largely focuses on preventative solutions to dejecta spillage, such as 3D‐printed ostomy flange stabilisers and ostomy bag sensors detecting pouch filling. Cosman et al. recognised that dejecta leakage may occur during ostomy bag reattachment; therefore, they developed an ostomy flange stabiliser, which serves as a firm backplate stabilising the extruding section of the stoma, known as a flange [118]. Similarly, the Ostom‐i Alert sensor from 11 Health was developed by an ostomate as a proactive measure to control leakage by receiving alerts about bag volume through a mobile application synced with the sensor [119]. Though these solutions may reduce the incidence of dejecta spillage, they do not provide an understanding or a solution to PSCs, and they are based on an assumption that all PSCs are due to spillage of dejecta, a hypothesis that remains unproven.

Moreover, solutions for dejecta spillage and PSCs are often reliant on human volunteer testing rather than in vivo or in vitro models, summarised in Table 4. Recently, Grove et al. sought to evaluate the effects of ostomy skin barriers to prevent leakage and irritation of the peristomal skin. Ostomy skin barriers are adhesive devices crafted to secure pouching systems onto the abdomen, safeguarding the peristomal skin from the dejecta. However, the study involved healthy volunteers without a stoma, excluding participants with insulin‐dependent diabetes, menopause, pre‐existing clinically significant skin diseases and damaged skin in or around the test site. All these exclusions are common characteristics of an ostomate; thus, the results of the study, while applicable to healthy Caucasians of Fitzpatrick skin type I, II, or III, may not be reflective of the wider ostomate population [120].

**TABLE 4:** A table summarising the in vivo and in vitro diseased skin models of relevance to PSCs, detailing the advantages and disadvantages of each approach.

Categorization		Skin model	Diseases modelled	Advantages	Disadvantages
In vivo		Murine	IAD [61, 93, 94, 95], peristomal dermatitis [96], psoriasis [106, 107, 108]	Cost‐effective to purchase and maintain; fast reproductive cycle.	Different body mass, GI tract length and wound healing mechanisms to humans; functional unit in the SI is void of mucosal folds and submucosa.
		Porcine	Superantigen‐induced dermatitis [105], IAD [102], allergic contact dermatitis [99], atopic dermatitis [103], psoriasis [109, 110]	Physiologically relevant (common anatomical structures of the SI lumen and skin).	Skin contains an overabundance of apocrine sweat glands; expensive purchase and maintenance; long reproductive wait‐time.
In vitro	2D	Monolayer	Psoriasiform cutaneous inflammation [73]	Simple; cost‐effective; reproducible.	Involves singular cell type; homogenous; propagated on flat environment.
		Co‐culture	Atopic dermatitis [74]	Simple; cost‐effective; easily determine interactions between more than 2 cell types.	Propagated on flat environment; cells mixed with no layered differentiation.
	3D	De‐epidermized dermis (DED)	Parakeratosis [77], psoriasis [78]	Displays human skin morphology; withstands culturing for up to 4–5 weeks; retains basement membrane antigens.	Lacks viable fibroblasts; ECM cannot self‐renew; requires excessive skin biopsies.
		Biomaterial‐based	Psoriasis [79], eczematous dermatitis [80], diabetes [82]	Increased customizabililty; controlled and replicable biophysical properties.	Limited shelf‐life; poor mechanical properties; animal collagen not representative of human.
		Self‐assembly	Psoriasis [85, 86], atopic dermatitis [85]	No need for exogenous material or skin biopsies.	Limited control over structure; require meticulous control over factors like pH, temperature, timing and concentrations.
	2D/3D	Skin‐on‐a‐chip (SOC)	Atopic dermatitis [89], oedema [90]	Combines favourable aspects of 2D and 3D models (lateral flow, multi‐layered depth); controlled microenvironment.	Generation and upkeep can be time‐consuming; requires microfabrication expertise.

On the other hand, utilising ostomates with active PSCs in product optimisation can be hazardous. Stoma care products were tested on 18 ostomates suspected of having peristomal dermatitis to identify products that serve as a PSC trigger. Rather than treating peristomal dermatitis, these tests further irritated the condition, with 12 out of the 18 subjects reacting to a product and experiencing pain and pruritus [121]. Another study tested ostomy pastes, particularly those with Gantrez ES‐425, on 26 patients suspected of having peristomal allergic contact dermatitis. Eighteen patients had one or more reactions but lacked concurrent treatment of dermatitis affecting the peristomal skin. Additionally, to expand on the notion that healthy individuals and ostomates are not easily comparable, patch tests conducted on ostomates using Gantrez ES‐425 revealed a reaction in 10 out of 13 cases. In contrast, none of the healthy volunteers tested with Gantrez ES‐425 displayed a reaction [122].

The ostomate population is complex, with distinctions between ostomates themselves—ileostomates, colostomates and urostomates. Ostomate surveys, including the Dialogue Study and the Ostomy Skin Study, have revealed that PSCs are more prevalent in ileostomates (57%–66%) than in colostomates (35%–57.2%) and urostomates (48%–60.9%) [9, 10, 113]. The difference in prevalence is hypothetically attributed to the stoma location and alterations in the active constituents of dejecta. An ileostomy is located more centrally on the right side of the abdomen, compared to a colostomy on the lower left side and a urostomy on the lower right side. Additionally, colostomates and urostomates excrete dejecta that are more reminiscent of their natural composition, respectively, faeces and urine. Ileostomates have digestive enzyme‐ and bile acid‐rich dejecta, more comparable to chyme than to faeces or urine. Moreover, in the GI system, the intraluminal pH drops (from 7.5 to 6.0–7.0) from the SI to the LI, as shown in Table 2. In the urinary system, the average pH of urine can vary between 5.5 and 6.5 [123]. These pH approximations can be reflected in dejecta; hence, leakage of colostomy and urostomy dejecta onto the skin may be less irritating than ileostomy dejecta, given that a more acidic pH is closer to the range of natural skin surface pH of 4.1 to 5.8 [33, 116].

Many ostomates also experience comorbidities that can accentuate the distinctions between healthy volunteers and ostomates. An ostomate likely has a GI disorder and possibly diabetes and/or obesity, which studies have shown are predisposing factors to PSCs. A retrospective study found a higher incidence of early post‐operative peristomal skin dermatitis reported among diabetic ileostomates [124]. Similarly, another study reported that overweight individuals with a BMI of at least 25 kg/m^2^ were roughly four times more likely to develop PSCs than non‐obese persons [112]. Pre‐existing skin conditions can also increase the risk of developing PSCs. In a study involving 232 ostomates, having psoriasis at baseline increased the risk of developing peristomal skin dermatitis by 33‐fold [114].

Healthy subjects cannot model the complexity of physiological changes induced by an ostomy, diabetes, obesity, or other disorders. However, utilising ostomates with active complications can aggravate and worsen their condition(s). Ultimately, researchers should strive to “do no harm,” and thus seek out alternative means to study PSCs. Developing PSC‐specific skin models would allow researchers to study the interplay between the active constituents of dejecta (bacteria, digestive enzymes and bile), while accounting for comorbidities and ostomy type. With diseased skin models existing for some of these comorbidities (diabetes, dermatitis and psoriasis), an ostomate‐specific model could, for example, induce dermatitis via a cytokine cocktail [85], before triggering a PSC, and testing ostomy products like skin barriers.

There is a wide range of in vitro and in vivo skin models that can be used to develop and refine such a model, especially 3D in vitro skin models which recapitulate the structure of the skin. Further development of these models could include the use of induced pluripotent stem cell (iPSC) technology to build disease‐specific or even personalised in vitro models of PSCs. iPSCs derived from patients with genetic skin conditions can be differentiated into relevant cell types and assembled into a 3D skin model representative of these conditions [125]. Arjmand et al. have differentiated human iPSCs into keratinocytes, allowing for personalised and patient‐specific models, and potentially treatments for vitiligo [126]. Similarly, iPSCs from patients or ostomates with conditions that predispose them to PSCs could be used to develop representative skin models to study PSCs and assess potential treatments that account for underlying conditions patients may have, e.g., psoriasis, diabetes etc. Using the wide range of cell differentiation protocols and microfluidic systems, vascularised and immune competent models could be developed [87, 127].

Hypothetically, the development of representative models of PSCs can also be compared with existing modes of PSC characterisation, like the Ostomy Skin Tool. The Ostomy Skin Tool is a standardised assessment for evaluating the severity and extent of PSC changes and could be used as a benchmark comparison during the development of such a model [128]. After inducing or integrating PSC‐like phenotypes into a skin model, the PSC could be scored by the Ostomy Skin Tool and then subjected to ostomy skin barrier testing to form a product efficacy vs. PSC severity curve.

Characterisation and understanding of the constituents of ostomy dejecta is critical to understanding its role in PSCs. Furthermore, the development of representative models of PSCs can aid in the elucidation of the mechanism behind PSCs and the identification of causative agents. This is an important step in the development of preventative technologies for PSCs, with implications for the development of improved treatments to enhance the quality of life for ostomates.

## Conflicts of Interest

Abram Janis is an employee and shareholder at Hollister Incorporated in Libertyville, Illinois. He serves as the industry partner on the Research Ireland Enterprise Partnership Scheme awarded to RCSI. Hollister Incorporated is the industry partner on both awards.

## Data Availability

Data sharing not applicable to this article as no datasets were generated or analysed during the current study.
